# Allele-specific analysis of DNA replication origins in mammalian cells

**DOI:** 10.1038/ncomms8051

**Published:** 2015-05-19

**Authors:** Boris Bartholdy, Rituparna Mukhopadhyay, Julien Lajugie, Mirit I. Aladjem, Eric E. Bouhassira

**Affiliations:** 1 Department of Cell Biology, Albert Einstein College of Medicine, 1300 Morris Park Avenue, Bronx, New York 10461, USA; 2 Laboratory of Molecular Pharmacology, Center for Cancer Research, National Cancer Institute, 37 Convent Drive, Bethesda, Maryland 20892, USA

## Abstract

The mechanisms that control the location and timing of firing of replication origins are poorly understood. Using a novel functional genomic approach based on the analysis of SNPs and indels in phased human genomes, we observe that replication asynchrony is associated with small cumulative variations in the initiation efficiency of multiple origins between the chromosome homologues, rather than with the activation of dormant origins. Allele-specific measurements demonstrate that the presence of G-quadruplex-forming sequences does not correlate with the efficiency of initiation. Sequence analysis reveals that the origins are highly enriched in sequences with profoundly asymmetric G/C and A/T nucleotide distributions and are almost completely depleted of antiparallel triplex-forming sequences. We therefore propose that although G4-forming sequences are abundant in replication origins, an asymmetry in nucleotide distribution, which increases the propensity of origins to unwind and adopt non-B DNA structure, rather than the ability to form G4, is directly associated with origin activity.

## Abstract (web summary)

DNA sequences contribute to the location and timing of replication origin firings. Here by allele-specific analysis, the authors show that replication asynchrony is associated with small cumulative variations in the initiation efficiency of origins, rather than with the activation of dormant origins.

## Graphical abstract

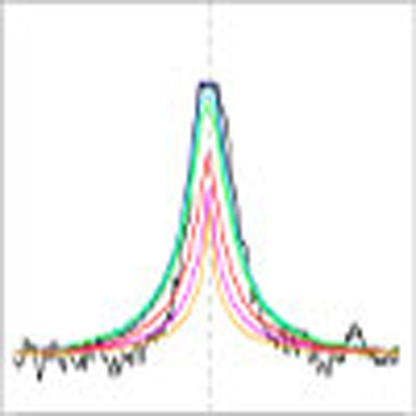

Mammalian DNA replication is a highly regulated process. Chromosomal regions rich in expressed genes tend to replicate early in S phase, while heterochromatin replicates later. The existence of this replication programme in mammalian cells was first demonstrated at the molecular level over 30 years ago through the study of specific gene loci^1^,^2^. Subsequent genome-wide analysis timing of replication using microarray and DNA-sequencing techniques^3^,^4^,^5^,^6^,^7^ revealed that the genome is organized in timing domains a few hundred thousand to a few million base pairs in size^7^,^8^. More recently, taking advantage of the decreasing cost of sequencing, we generated higher-resolution maps of timing of replication in human primary basophilic erythroblasts. These 50-kb resolution maps revealed that the previously characterized timing domains are composed of subdomains that we termed timing ripples^9^. These timing ripples are caused by groups of origins of replication and are highly reproducible between individuals.

The temporal regulation of the timing programme can also be studied by comparing the timing of replication of the two chromosome homologues. Using a genome-wide allele-specific approach based on the study of primary erythroid cells from individuals whose genome has been completely sequenced and phased^10^, we demonstrated that the two homologues replicated at the same time in ∼91.5% of the genome. A fraction of the 8.5% of the genome that replicated asynchronously was associated with parental imprinting and with the presence of large deletions. Similar studies by the Koren group suggested that the inactive X chromosome and heterochromatic regions were less tightly regulated than the rest of the genome^11^,^12^.

Remarkably, this strict regulation of the timing of DNA replication is not associated with a comparably strict regulation of the location of origins of replication, as can be demonstrated by analyses of stretched DNA molecules^8^,^13^,^14^ or by the analysis of nascent strand (NS) synthesis^13^,^14^,^15^,^16^. Many studies have shown that, at the single-molecule level, the initiation of DNA replication does not occur at the same position in every cell. Rather, although initiation occurs preferentially in zones of replication, individual origins of replication are used only in a fraction of the cells. The decision of which origin will be used during S phase is apparently stochastic. The mechanisms underlying this stochasticity are not completely understood but likely reflect competition between DNA replication and transcription^17^,^18^,^19^. The strict regulation of the timing of replication that can be detected at the 50–100-kb scale as timing ripples therefore reflects the average activity of origins of replication that are stochastically utilized at the molecular level.

The molecular mechanisms that control the origin location and the timing of their firing remain imperfectly understood. Autonomously replicating sequences define replication origins in yeast but no consensus sequences have been found in mammalian cells^20^. Sequencing and microarray studies in *Drosophila*, mouse and human cells have detected more than 300,000 sequences that are enriched in newly synthesized NSs and that are therefore considered to be origins of replication. Sequence analysis revealed that these origins of replication are enriched in transcription start sites and CpG islands, confirming earlier studies^21^. In addition, the most intriguing observation from these sequencing studies was that the origins of replication are greatly enriched in sequences that have the potential to form G-quadruplexes (G4)^9^,^22^,^23^.

G4s are DNA secondary structures that form when four consecutive triplets of DNA interact by Hoogsteen pairing^24^. G4s can readily be detected *in vitro* and probably form *in vivo*, notably at telomeres^25^. The association between G4-forming sequences and putative origins of replication was unexpected because previous work suggested that G4s were associated with fork stalling rather than with initiation of replication^26^,^27^,^28^,^29^. Nevertheless, a recent study based on site-directed mutagenesis suggested that there might be a causal relationship between the presence of G4s and origin activity^30^.

To determine the genome-wide significance of the association between NS peaks and G4s as well as other sequences known for potential of secondary structure formation, and to elucidate the mechanism of generation of asynchronous timing regions, we have performed allele-specific NS sequencing experiments and a detailed analysis of origin sequences. We found that the presence of G-quadruplex-forming sequences does not correlate with the efficiency of initiation. Sequence analysis revealed that the origins are highly enriched in sequences with profoundly asymmetric G/C and A/T nucleotide distributions and are almost completely depleted of antiparallel triplex-forming sequences. We propose that the propensity of origins to unwind and adopt non-B DNA structure, rather than the ability to form G4, is associated with origin activity.

## Results

### Generation of allele-specific replication origin profile

**Figure 1:**
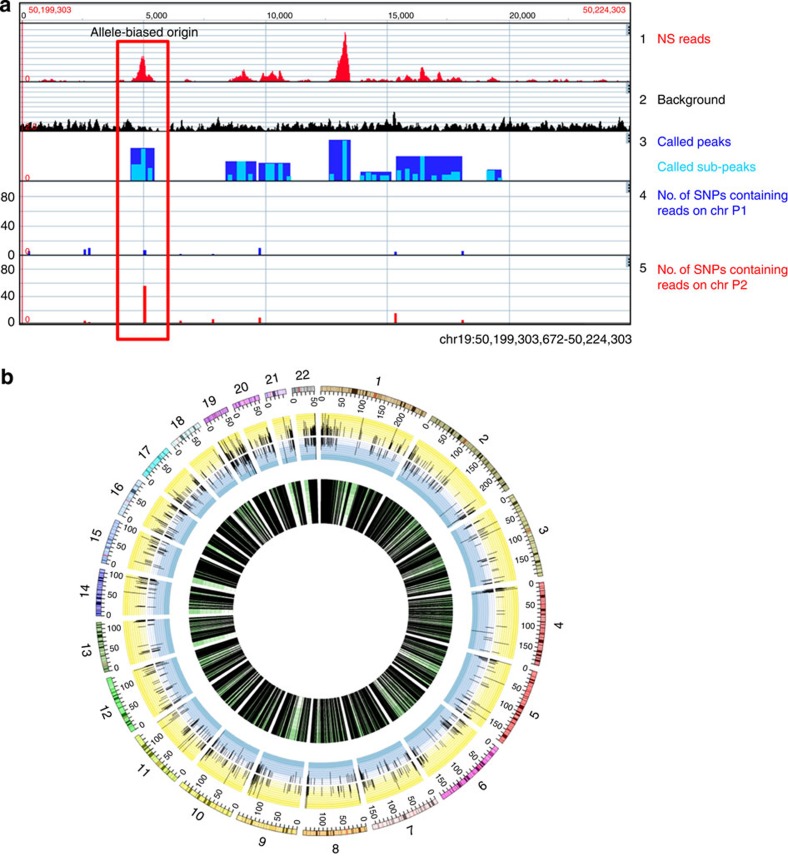
Allele-specific NS analysis. (**a**) GenPlay genome browser screenshot illustrating allele-specific NS sequencing analysis. Track 1: uniquely aligned NS reads binned in 10-bp windows. Track 2: background track obtained by sequencing DNA from control white blood cells from same individual. Track 3: peaks (dark blue) and subpeaks (light blue) called using the MACS software. Tracks 4 and 5: allele depth of phased heterozygous SNPs in FNY01_2_2 in chromosomes P1 (track 4) and P2 (track 5). The red rectangle highlights an allele-biased origin of replication (see text). (**b**) Circos plot illustrating the location of all allele-biased origins. Green (inner) circle: location of all origins analysable in an allele-specific manner. Outer circle shows the log2 ratio of P1/P2 reads in allele-biased origins (blue, negative; yellow, positive values).

To obtain allele-specific profiles of replication origins, we generated 98% pure populations of highly proliferative primary erythroblasts by culturing peripheral blood haematopoietic stem and progenitor cells from FNY01_2_2, an individual whose genome was completely sequenced and phased^10^. These erythroblasts were pulsed with BrdU for 30 min, short single-stranded DNA fragments were purified by sedimentation and newly synthesized NS were immunoprecipitated with anti-BrdU antibodies. Sequencing of the newly synthesized NSs yielded about 184 million reads uniquely aligned to the human genome. Regions enriched in NS were then called with the MACS peak-calling software^31^ (Fig. 1a). This yielded 164,347 regions that displayed at least a fivefold enrichment over control with a false detection rate (FDR) of 0.01. The median size of these regions was 772 bp and the upper and lower quartiles were 485 and 1,285 bp, respectively. Some of the regions thus defined contained two or more closely spaced subpeaks and therefore a list of 456,000 subpeaks was generated for further analysis. This new high-read-depth NS profile correlated strongly with a previously obtained lower read-depth NS profile^9^ since 95% of the top 50,000 peaks observed in the low read depth profile overlap with a peak in the high-read-depth profile.

To measure the origin efficiency in an allele-specific manner, we used the GATK software^32^ to call the single-nucleotide polymorphisms (SNPs) and small insertion and deletions (indels) in the NS sequencing data and the GenPlay Multi-Genome software^33^ to assign all SNP-containing reads to the two parental chromosomes, arbitrarily termed P1 or P2, using the phasing information that we previously generated. We then computed for every NS peak the ratio of the number of reads that could be specifically assigned to chromosomes P1 or P2. This ratio is a direct measure of origin efficiency for each origin on the two chromosome homologues.

NS peaks containing at least 50 allele-specific reads were then selected for further study. A total of 22,844 origins out of about 164,000 (13%) fit that criterion and were deemed analysable in an allele-specific manner (Table 1). To determine whether the genomic distribution of the subset of analysable origins was similar or different from that of all origins, we computed the frequency of G4, TSS, CpG Islands, SINE, LINE, LTR and imprinted genes for both groups of origins. This analysis revealed that the analysable origins were enriched in most of the tested features (Table 1). Further analysis revealed that the analysable origins were on average stronger than the bulk of the origins since, by definition, they contained at least 50 reads. To test whether the extent of enrichment was similar to the enrichment observed in a comparable group of replication origins, we created a new origin subgroup by resampling the non-analysable origins to obtain a distribution of peak heights that was the same for the analysable and non-analysable origins. The proportion of G4, imprinted genes, SINE and LINE observed in the resampled replication origins was similar to that observed for the analysable origins. We concluded from this analysis that the results obtained from the subset of analysable origins can, in first approximation, be extrapolated to all relatively strong origins that we detected in the genome.

Binomial tests after correction for multiple testing revealed that 1,992 origins (8.9% of the analysable origins) had significantly different number of reads on the two homologues at an FDR of 0.05, and were therefore allele-biased origins of replication. The ratio of origin usage by the two homologues ranged from about 1.5 to more than 100 (Supplementary Fig. 1). The most active alleles of each pair of origins were about equally distributed on the P1 and P2 chromosomes (Fig. 1b). To determine whether the genomic distribution of the subset of allele-biased origins was different from that of all analysable origins, we repeated the analysis described above for these two subsets of origins. As shown in Table 1, the distribution of the allele-biased origins for all the tested genomic features was very similar to that of the bulk of all analysable origins, suggesting that there was no obvious selection for allele specificity for any of the tested genomic features.

### Asynchronous regions are enriched in allele-biased origins

We previously defined, in two individuals, about 600 asynchronously replicated domains (ARDs) that had an average size of 1.5 Mb and an average asynchrony of about 48 min as compared with <15 min for the entire genome^9^. We also defined core ARDs representing the most asynchronous regions within the ARDs using a more stringent peak detection cutoff (see methods). The average size of these core ARDs was about 200 kb and their average asynchrony was 68 min.

**Figure 2:**
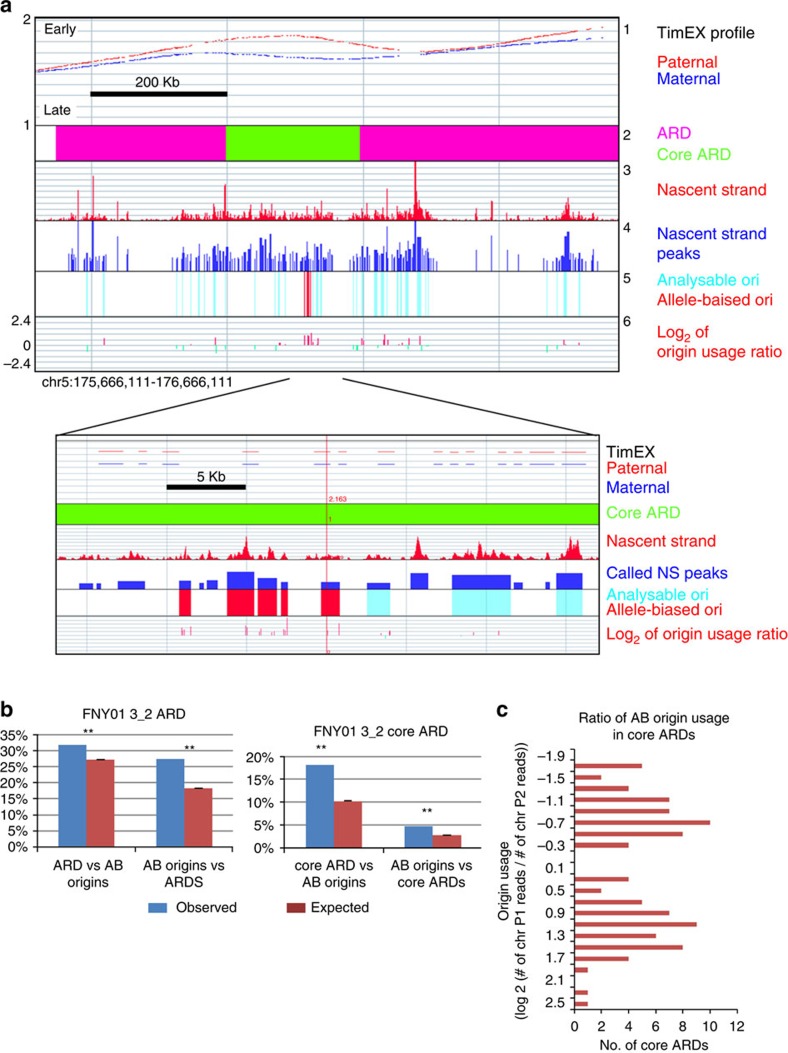
Asynchronously replicated regions (ARDs) are enriched in allele-biased origins of replication. (**a**) Track 1: timing of replication profile of a genomic region containing a 1-Mb ARD. The blue and red curves, respectively, represent the TimEX profiles of the maternal and paternal chromosomes. The *y* axis represents the S/G1 TimEX ratio, which is proportional to the replication time during S phase. The TimEX ratio is the ratio of the number of reads observed in the S and G1 phase of the cell cycle and was calculated in 5,000-bp windows genome-wide. Track 2: pink, ARD; green, core ARD. Track 3: NS profile (binned in 10-bp windows). Track 4: NS peaks called by MACS. Track 5: light blue: analysable origins (that is, origins overlapping at least 50 SNP-containing reads); red: allele-biased origins (origins with statistically different number of reads on both alleles (FDR<0.05)). Track 6: log ratio of the number of reads observed in P1 and P2. Zoomed-in region: same as above but magnified 40 times. (**b**) ARDs and core ARDs are enriched in allele-biased origins of replication. Left histograms: per cent ARDs that contain allele-biased origins. Right histograms: per cent core ARDs that contain allele-biased origins (AB origins). Blue bars, observed values; red bars, expected values. Stars indicate that the differences between observed and expected values were significant (permutation *P* value <0.001). Expected values and *P* values were calculated by performing 10,000 permutations. Error bars represents s.e.m of permutations. (**c**) Histogram illustrating the distribution of allele bias in origins located within core ARDs. *x* axis, number of core ARDs; *y* axis=log_2_(number of reads on chr P1/number of reads on chr P2).

Conceptually, timing asynchrony can be caused either by differential efficiency of replication origins or by the activation/licensing of additional, dormant origins. We addressed the first possibility by calculating the percentage of allele-biased origins of replication that overlapped with ARDs and core ARDs in the two TimEX data sets that we generated previously and performed permutation tests to assess statistical significance (Fig. 2a,b). About 31.8% of the ARDs overlapped with at least one allele-biased origin, while only 27.1% were expected by chance (permutation *P* value=0.002, Fig. 2b left panel). As expected, the enrichment was more dramatic for the core asynchronous regions with 18.1% of the core ARDs overlapping with at least one allele-biased origin, while only about 10.21% would have been expected by chance (permutation *P* value <10E−4, Fig. 2b, right panel). Similarly, 27.4 and 5.9% of the allele-biased origins were, respectively, within ARDs and core ARDs, while 18.3 and 2.9% were expected by chance (permutation *P* value <10E−4 in both cases). Very similar results were obtained when data from individual FNY01_3_3 were analysed (Supplementary Fig. 2A,B). As a control, we then tested the enrichment of ARDs in non-allele-biased origins. As shown in Supplementary Fig. 2C, the ARDs were not enriched in non-allele-biased origins. Together these results strongly suggest that replication asynchrony is associated with changes in origin efficiency between the two alleles.

To address the second possibility and to determine whether new origins were activated in asynchronous regions, we plotted the origin usage for all allele-biased origins overlapping the ARDs or the core ARDs (Fig. 2c). This revealed that the vast majority of AB origins were active on both alleles but were two to three times more active in one allele, suggesting that the main mechanism by which replication timing asynchrony is generated is modulation of origin activity rather than activation of dormant origins.

### G4-forming sequences do not correlate with origin usage

The most striking observation that came out of previous analyses of NS-sequencing experiments was that G4s are enriched at origins of replication. This exciting result suggested that these sequences might be essential for the regulation of origin activation.

To test this hypothesis, we asked whether SNP or indels that disrupt or create G4-forming sequences affect origin efficiency. To perform this analysis, we identified all allele-specific G4-forming sequences in allele-biased origins present in individual FNY01_2_2 using the QuadParser 2.0 programme^34^. We found 4,393 G4-forming sequences in the 1,992 allele-biased origins of replication. Ninety-four of those G4-forming sequences, located in 86 origins of replication, contained heterozygous SNPs or indels that disrupted their ability to form G4s.

**Figure 3:**
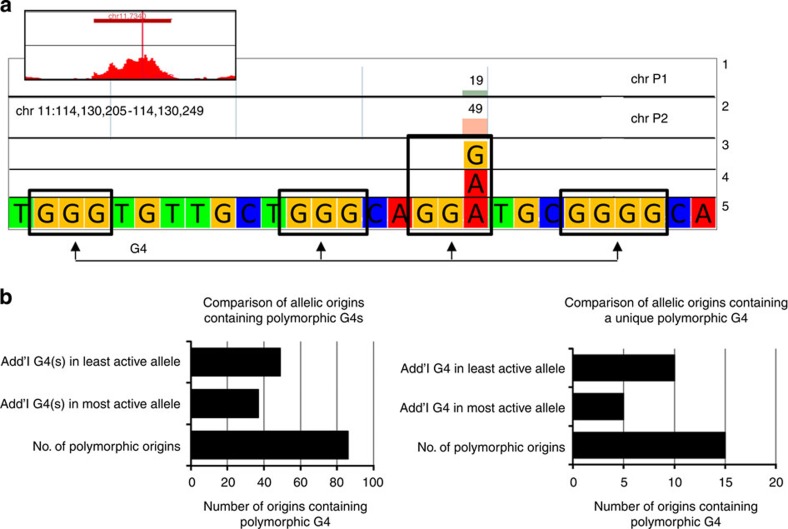
Number of G4s in allele-biased origins does not correlate with origin usage. (**a**) A G4 quadruplex at position 114,130,205–1,114,130,249 on chr 11 is present on chr P1 but not on chr P2 because of a G to A transition that destroys the third triplet of Gs. Tracks 1 and 2 illustrate the number of reads containing a G or an A at that position. Tracks 3 and 4 illustrate an SNP that alters a G4-forming sequence. Track 5 illustrates the sequence of the G4. Black rectangles highlight the four triplets of G and the G to A SNP. (**b**) Presence of G4s does not correlate with origin activity. Left histogram: *x* axis, number of origins containing one or more polymorphic G4. Right histogram: same but restricted to origins containing at most 1 G4. Additional G4s created by SNPs or indels are about equally distributed between the least and most active alleles.

To determine if the polymorphic G4-forming sequences affected DNA replication, we compared origin usage with the number of G4-forming sequences in each of these pairs of allelic origins. The allele containing one or more additional G4-forming sequences was the most efficient origin in 37 out of 86 cases and the least efficient in the remaining 49 cases (Fig. 3a and Table 2). Polymorphic G4-forming sequences were therefore associated with decreased origin usage in the majority of cases.

However, since many origins contain more than one G4-forming sequence, it was possible that this redundancy was masking the effect of individual sequences. We therefore repeated the analysis but compared only the pairs of allelic origins that contained a single polymorphic G4-forming sequence (Fig. 3b and Table 2). Fifteen allele-biased origins fell in that category. The allele containing the G4-forming sequence was the most efficient in five cases and the least efficient in 10 cases confirming that the presence of a G4 is not necessary for origin formation and is generally associated with decreased origin efficiency.

**Figure 4:**
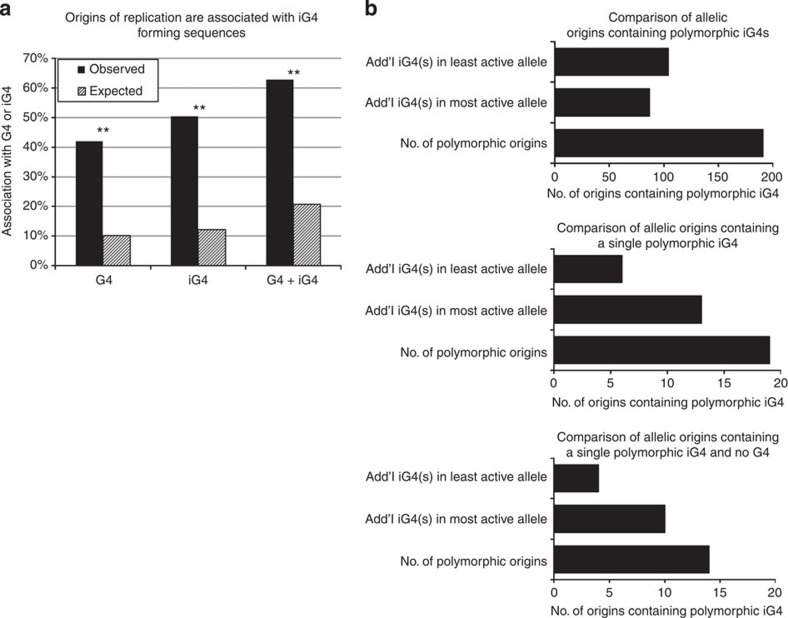
Intermolecular iG4s are highly enriched in origins of replication but their presence does not correlate with origin usage. (**a**) Origins of replication contain more iG4s than expected by chance. Bar plot illustrating the association between G4s, iG4s and origins of replication. *y* axis, per cent of origins associated with G4s, iG4s or both. Black bars: observed association, striped bars: association expected by chance (10,000 permutations). Stars indicate that the differences between observed and expected values were significant (permutation *P* value <0.001). (**b**) Presence of iG4s does not correlate with origin activity. Top plot: *x* axis, number of origins containing one or more polymorphic iG4. Middle plot: same as above but restricted to origins containing at most 1 iG4. Bottom plot: same but restricted to origins containing no G4s and at most one iG4.

Since G4s can also form intermolecularly^35^,^36^, we searched the genome for all intermolecular G4-forming sequences (iG4) that we defined as sequences that contain four consecutive triplets of either Cs or Gs separated by no more than seven nucleotides. We found 485,926 iG4s in addition to the 361,000 G4s in the human genome. Importantly, overlap analysis revealed a very strong, highly significant association between origins of replication and iG4-forming sequences (Fig. 4a). However, the analysis of 191 pairs of allelic origins containing different numbers of iG4s due to the presence of SNPs or indels revealed that, as for G4-forming sequences, iG4-forming sequences were not preferentially associated with the most efficient origins (Fig. 4b).

### Origins are GC rich independent of G4-forming potential

Since neither G4- nor iG4-forming sequences seem to regulate the origin efficiency, we asked whether the association between the origins of replication and G4-forming sequences was secondary to enrichment in GC-rich regions.

**Figure 5:**
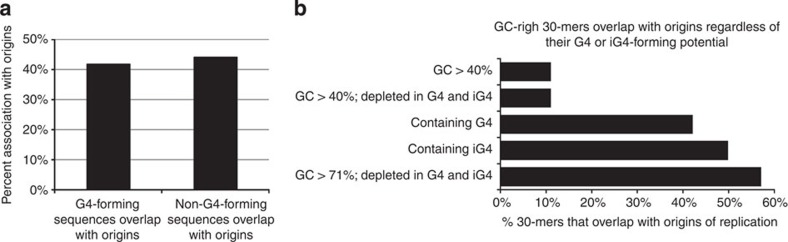
Origins of replication are associated with GC-rich regions independently of their G4-forming potential. (**a**) Control sequences with the same length distribution and the same GC-content as G4- and iG4-forming sequences are as strongly associated with origins as G4- or iG4-forming sequences. (**b**) Origins of replication are associated with GC-rich 30-mers independently of their G4-forming potential. Bar plots illustrating the per cent overlap between 30-mers with GC-content >40% (top bar), 30-mers with GC-content >40% depleted in G4s and iG4s (second bar), 30-mers containing G4s or iG4s (third and fourth bars), 30-mers with GC-content >71% depleted in G4- and iG4-forming sequences.

To address this question, we excluded all G4- and iG4-forming sequences from the hg19 genomic sequence and selected random regions with the same length and the same GC-content distribution as G4 and iG4-forming sequences. This analysis revealed that GC-rich regions that have no potential for intra- or intermolecular quadruplex formation were as strongly associated with origins of replication as G4-forming sequences since 44% of the origins overlapped with at least one non-G4-forming GC-rich control sequence, while 42% of the origins overlapped with at least one G4-forming sequence (Fig. 5a).

A second analysis based on the comparison of all the 30-mers that had similar GC-content but that contained or did not contain G4- and iG4-forming sequences revealed that 57% of high GC-content 30-mers that could not form G4 or iG4-forming sequence overlapped with an origin of replication, while only 42.2 and 49.9% of the 30-mers that contain G4- and iG4-forming 30-mers overlapped with an origin of replication (Fig. 5b). GC-rich 30-mers that do not contain G4- or iG4-forming sequences were therefore more strongly associated with origins than similar 30-mers with this potential.

These analyses suggest that the enrichment in G4- and iG4-forming sequences is secondary to the high GC-content of origin of replication and confirm that the presence of G4-forming sequences is not the main determinant for origin formation.

### Origins are profoundly G/C and A/T skewed

**Figure 6:**
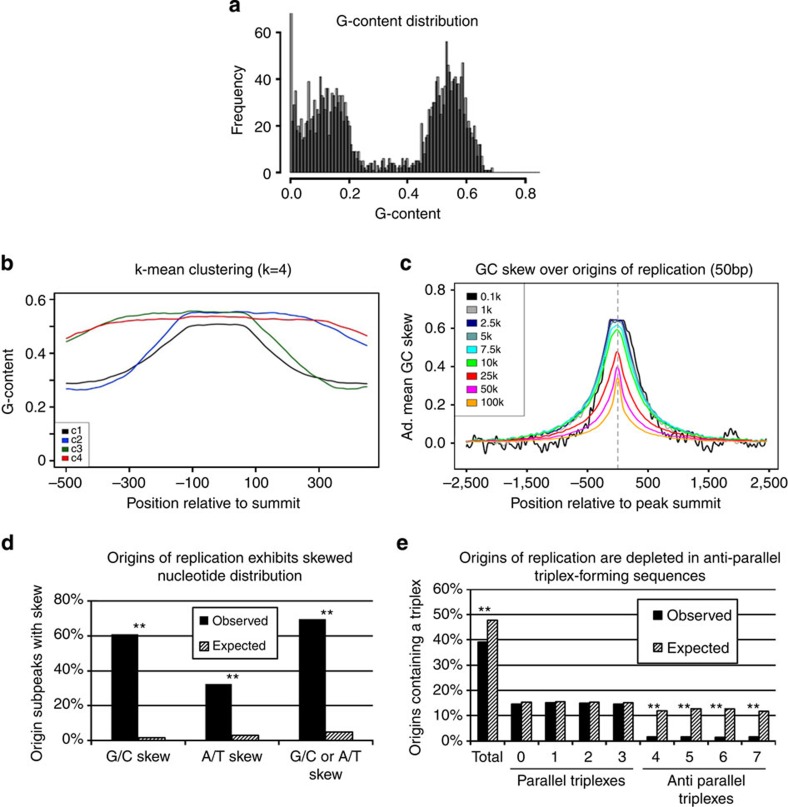
Origins are strongly skewed and depleted in antiparallel triplex-forming sequences. (**a**) Histogram illustrating G-density in origin subpeaks. Mean density on the reference allele are shown for the top 3,000 origin subpeaks. The bimodal distribution reflects the fact that the G-rich strand is either on the plus or minus strand. Origins have a highly biased G- and C-content. (**b**) *k*-means clustering of G-density (*k*=4) in origins of replication. The size of the G-rich region varies from about 200 bp to 1 kb. The 200-bp regions at the centre of the top 3,000 origin subpeaks are G-rich (on either the plus or minus strand). Many origins exhibit much longer G-rich regions. (**c**) Density plots of G/C skew in origin subpeaks stratified by efficiency. Black curve, adjusted G/C skew of the top 100 most efficient origins (0.1 k). Grey line: adjusted G-density for the top 1,000 origins (1 k) and so on. G/C skew was adjusted by reverse complementing the origins in which the G-rich strand was the minus strand (see methods). Highly efficient origins contain large 200–500-bp skewed regions. The size and the amount of skew progressively decrease in less-efficient origins. *y* axis=GC skew (G-C/G+C). (**d**) Histogram depicting the proportion of skewed origins. Top 10,000 origin subpeaks were analysed by intersecting the origin subpeak with a track containing all G/C or A/T skewed regions in the human genome that are greater than 400 bp. Expected values were calculated by permutations. Stars indicate that the differences between observed and expected values were significant (permutation *P* value <0.001). (**e**) Histogram illustrates the per cent origins of replication that contain a triplex-forming sequence. Triplex-forming sequences were detected using Bioconductor package Triplex and intersected with origins of replication. Types 0–3 are parallel triplex, while types 4–7 are antiparallel. Antiparallel triplexes are found in origins <10 times as often as would be expected by chance. Stars indicate that the differences between observed and expected values were significant (permutation *P* value <0.001).

Since Cayrou *et al*.^22^,^37^ reported that the origins of replication were enriched in 20–30 bp loose G-rich motifs, we computed the G-content of 100-bp windows centered on the summit of the 3,000 most-enriched origin subpeaks. Analysis of the distribution of G-content in these origins yielded a strongly bimodal curve with two peaks of about equal size at about 15 and 55% G-density (Fig. 6a). This demonstrated that this set of origins had a very pronounced G-density asymmetry and that the G-rich strand could be either the plus or the minus strand. Extension of this analysis to weaker origins revealed that the distribution of G-content was progressively less biased as the origin efficiency decreased (Supplementary Fig. 3). To further characterize the distribution of G-density, we reverse complemented the origins with a low G-content on the plus strand and performed a *k*-means clustering analysis (Fig. 6b) to determine if these origins could be classified in subcategories based on their G-content. This revealed that almost all origins in the group of highly efficient top 3,000 origins share a large 200–500-bp G-rich region centred on the summit of the NS peaks. To confirm these observations, we stratified the origin subpeaks by strength and plotted their G/C skew. This revealed that the strong origins of replication are profoundly G/C skewed and that the skewed regions decrease in size as origin efficiency decreases (Fig. 6c and Supplementary Fig. 4). Importantly, plotting the A/T skew revealed that a fraction of the origins were A/T rather than G/C skewed (Supplementary Fig. 4) suggesting that the presence of a skew rather than a specific sequence was the important characteristic. To determine the percentage of strong origins that exhibit a skew, we called all the regions that displayed a skew genome-wide, and overlapped the results with the origin subpeaks. About 61% of the strongest 10,000 subpeaks were G/C skewed, 32% were A/T skewed and 70% were either G/C or A/T skewed (Fig. 6d). Permutation analysis demonstrated that these associations were highly significant (permutation *P* value <0.001).

### Depletion of triplex-forming sequences in origins

Purine-rich regions, such as the skewed regions present in origins, can form DNA triplexes that are non-B-form DNA structures, which contain a third strand that is stabilized via Hoogsteen or reverse Hoogsteen bonds^38^. Several types of triple helixes can form depending on the orientation of the third strand with respect to the purine-rich strand^39^. Types 0–3 are parallel triple helices, while types 4–7 are antiparallel. Analysis using the Triplex package from Bioconductor revealed a profound depletion in triplex-forming sequences in origins of replication (Fig. 6e). Importantly, this depletion was specific for antiparallel triplexes, which suggested biological significance because antiparallel triplexes are much more likely to form *in vivo* than parallel triplexes since the latter types of triplexes only form at low pH and after protonation.

To determine if we could detect any effect of triplex presence on origin usage, we performed an allele-specific analysis similar to the one performed previously for G4- and iG4-forming sequences. We observed that all triplexes that were created or destroyed by SNPs or indels were of the parallel type. There were 26 cases in which pairs of origins contained one allele harbouring a single triplex-forming sequence, while the other allele had none. In 12 cases, the triplex was associated with the most active origin, in 14 with the least active. These data suggest that sequences that can form a parallel triplex have no effect on origin usage. No antiparallel triplexes were created by SNPs or indels in the 1,992 allele-biased origins tested.

## Discussion

Analysis of over 184 million pair-ended reads allowed us to measure the origin efficiency in an allele-specific manner for about 22,000 origins, 13% of all detected origins. Differential origin usage between the two homologues was detected in 8.9% of these 22,000 origins. Therefore, although origin usage is stochastic, 91.1% of all origins are used by both homologues.

Importantly, we demonstrate for the first time that small changes in initiation efficiency, rather than the activation of dormant origins, accounts for the replication asynchrony associated with imprinting and the presence of large deletions. This contrasts with the activation or inactivation of dormant origins of replication that is associated with tissue-specific changes in timing of replication^40^ and with the cellular response to DNA damaging conditions^41^ suggesting that different mechanisms might be at play.

A recent report based on the analysis of lymphoblastoid cell lines sequenced by the 1,000 Genomes project revealed the existence of replication timing quantitative traits that are associated with inter-individual variation in timing suggesting that replication timing can be genetically determined^42^. Interestingly, 10 out of the 20 regions affected by a replication timing quantitative trait locus in lymphoblastoid cells were identified as ARDs in our study, an association that is much greater than would be expected by chance (permutation *P* value <10.E−5). This suggests that the replication timing variants identified as replication timing quantitative trait loci might also be associated with small changes in initiation efficiency at multiple origins.

Analysis of allele-specific origin efficiency profiles has allowed us to determine whether sequence changes that disrupt G4- or iG4-forming sequences alter the efficiency of more than 250 origins in their natural locations. This novel approach, which is equivalent to having performed 250 knock-in experiments in human primary cells, clearly demonstrated that G4- or iG4-forming sequences have no detectable effect on origin efficiency. This conclusion was supported by the observation that the enrichment in G4- and iG4-forming sequences was secondary to the high GC-content of origins of replication.

Our results are in agreement with studies based on genetic dissection of individual origins that showed that an asymmetric GC-rich content, but not G4 sequences, were essential for initiation^43^,^44^, but are in apparent contrast with the results of Valton *et al*.^30^, showing that disruption of two G4s dramatically affected origin usage. The two findings are, however, not incompatible since the latter results were based on experiments in which the potentially G4-forming sequences were replaced by AT-rich sequences, and therefore did not explicitly demonstrate that the observed changes in origin usage were directly caused by the lack of G4 formation. Hence, it was possible that the changes observed by Valton *et al*. were caused by another mechanism not related to physical formation of a quadruplex. The enrichment of potential G4-forming sequences in replication origins might therefore reflect their high GC-content and asymmetry, not an essential property required for initiation.

The lack of effect of G4-forming sequences on origin usage led us to reanalyse the origin sequences. This revealed two important characteristics. Origins of replication are almost completely depleted in sequences that can form antiparallel triplexes, and most origins have a profound asymmetry between the two strands. Importantly, we found a strong correlation between the length of the skewed regions and origin efficiency suggesting that the skewness of the sequences might be functionally important.

The depletion in antiparallel triplex-forming sequences suggests that such sequences might be incompatible with initiation of replication. Several reports have shown that triplex-forming regions can stall replication forks^45^,^46^. Kim *et al*.^47^ reported that the presence of a triplex-forming sequence stalled replication forks in an orientation-dependent manner because purine-rich DNA is a poor substrate for DNA polymerase α (Polα), the polymerase that synthesizes Okazaki fragments. The depletion in triplex-forming sequences in origins of replication might therefore reflect the fact that both the leading and lagging strands are primed by Polα during initiation of replication.

It was previously reported first in prokaryotes, and later in eukaryotes, that DNA sequences on either side of origins of replication are skewed with a prevalence of G over C and T over A on the leading strand^48^. These imbalances can be observed up to several hundreds of kb from the origins and are believed to reflect differential mutational pressure on the lagging and leading strands in germ cells. The sequence skews that we detected in the 1-kb regions surrounding the centre of most NS peaks are much stronger than the long-range skew detected earlier and is not polarized relative to the direction of replication. Origin sequences are strongly skewed but the G-rich or A-rich strand can be either the positive or the negative strand. The evolutionary mechanisms that led to the formation of these two types of skews are therefore likely to be different.

DNA regions with asymmetric G/C and A/T nucleotide distributions are found in rDNA, minisatellites and hypervariable regions and are enriched in regulatory regions such as promoters, immunoglobulin switch regions and telomeres. One important characteristic of these skewed regions is that they can form R or D loops. It was recently shown that R-loop formation in G-rich regions is a distinctive characteristic of unmethylated human CpG island promoters^49^. Intriguingly, the O_H_ origin of replication in human mitochondrial DNA is located in a D-loop^50^ and has a G-density profile that is very similar to many of the strongest nuclear origins (Supplementary Fig. 5). Therefore, mitochondrial and nuclear origins of replication share similar DNA sequence characteristics.

In accordance with our data, a recent study showed that the origins of replication in several yeast species are often low-complexity nucleosome-excluding regions^51^. DNA regions with asymmetric G/C and A/T nucleotide distributions can form strong stacking interactions that introduce distortion in the DNA double helix and lead to the formation of non-B DNA with a greater tendency to unwind than B-form DNA^52^, which might favour binding of the origin replication complex and initiation of replication. Factors that recognize these non-B DNA structures might also contribute to origin recognition.

## Methods

### Human subject

Blood samples from individual FNY01 2_2 was obtained with informed consent under Einstein IRB approved protocol number 2011-356.

### Cell culture

Peripheral white blood cells (10–20 ml) were harvested by venipuncture from individuals from family FNY01 under an approved IRB protocol. Mononuclear cells were isolated by density gradient centrifugation on Histopaque (Sigma-Aldrich) according to the manufacturer's instructions. The purified cells were frozen in two million cell aliquots. Two million mononuclear cells were expanded and differentiated into basophilic erythroblasts in culture for 2 weeks in serum-free StemSpan media (Stem Cells Technologies, VA, CA) containing the cytokine cocktail mix described by Olivier *et al*.^53^. At the end of the culture, cells were immunophenotyped by fluorescence-activated cell sorting using antibodies against CD71 (e-Bioscience 11–0719, 0.3 mg ml^−1^) and CD235a (e-Bioscience 11–9987, 0.6 mg ml^−1^). Cells were relatively uniform in size and more than 97% of the cells were double-positives demonstrating that the vast majority of cells in the culture were erythroid cells at the basophilic stage of differentiation.

### Nascent strands

Nascent, newly replicated DNA strands (NS) from cultured basophilic erythroblasts were isolated based on incorporation of the nucleotide analogue BrdU as described by Aladjem *et al*.^54^. In brief, we labelled the cells with a 30- min pulse of BrdU, lysed the cells and fractionated small DNA strands (<1 kb) using a neutral sucrose gradient. We then employed immunoprecipitation with anti-BrdU antibodies (BD Pharmingen, cat. no. 555627, 0.5 mg ml^−1^) to isolate newly replicated DNA strands on the basis of selective incorporation of BrdU. The resulting NS were subject to massively parallel sequencing on a single lane of an Illumina HiSeq 2500 using the standard Illumina protocol. Sheared genomic DNA was sequenced as a standard to control for mapability and other potential biases.

### Alignment

Pair-ended 150 bp reads (252,816,875) were obtained and aligned to hg19 using bwa or Bowtie2 using the default settings^55^. Both aligners gave very similar results. Alignment with Bowtie2 resulted in 184,819,425 mapped reads. Mapped reads were loaded in GenPlay in bins of 100, 3 or 20 kb (depending on the analysis).

Because the NS library that we generated was not strand specific, the read density was expected to be highest at the centre of the origins. Peaks were called using MACS 2.010 using a *q* value cutoff of 0.05 and shift size 250, with or without the additional call-summits option to call subpeaks^31^. Data were obtained from sequencing DNA from white blood cells of individual FNY01_2_2 as a control (335,897,106 reads, of which 320,142,920 mapped). A file containing 164,000 peaks that had at least a fivefold enrichment over the control was generated as well as a file in which these peaks were split into subpeaks.

### Allele-specific analysis

To generate allele-specific TimEX profiles, SNPs and indels were called with the GATK variant caller in the known-allele mode^32^, using the vcf file that describes the phased genomes of family FNY01 (ref. 10) as a reference file. In this mode, the GATK calls only the SNPs at the positions specified in the user-provided reference vcf file.

Once the variants had been called, the vcf files for the NS library were phased using the vcf for family FNY01 as a reference and specific functions in GenPlay multi-genome^56^. Once the files were phased, files (in BED format) containing the allele depth for the paternal and maternal chromosomes were generated, again using specific functions provided in GenPlay.

Origin usage for each of the 164,327 called peaks (origins of replication) was then computed by summing the read depth of all the SNPs contained in each of the peak for the two parental alleles, in the following termed P1 and P2. The rbinom and the p.adjust R functions were then used to perform a binomial test and calculate the statistical significance of the difference of origin usage between the P1 and P2 chromosomes. Origins containing <50 reads specific for P1 and P2 were filtered out. An FDR of 0.05 was set for the p.adjust function. This analysis resulted in the discovery of 1,992 allele-biased origins of replication in the genome of FNY01_2_2. Although we also called indels, this analysis was restricted to SNPs to eschew the significant error rate associated with indel calling at the given coverage and read length.

### Definition of ARDs and core ARDs

TimEX data used in these studies was previously published and can be accessed under accession number GSE50978. The methods used to call the region subject to differential timing are described in details in ref. 9. In brief, asynchronously replicated regions were detected with the GenPlay island finder on a data file generated by subtracting the paternal S/G1 simulated profiles from the maternal S/G1 simulated profiles. The GenPlay Island finder function is based on the SICER algorithm of Zang *et al*.^57^ and identifies broad regions of enriched read counts rather than peaks. The algorithm allows for gaps in the island and is well suited for the allele-specific TimEX data because of the heterogeneous distribution of the SNPs in the human genome. Implementation of the algorithm in GenPlay is described in the GenPlay documentation on the GenPlay web site.

A TimEX difference threshold of 0.02, a maximum gap size of 250,000 bp and a minimum island size of 50,000 bp were used in the island finder. Statistically significant regions were then identified using a *χ*^2^-test for goodness of fit (chisq.test function in R) on 2 × 2 contingency tables built by calculating the total number of reads obtained in the S and G1 fraction of the maternal and paternal chromosomes for each island. *q* values were then calculated using the p.adjust function in R (with the fdr parameter) to control for multiple testing. An FDR of 5% was used to define the statistically significant asynchronous replication domains. The parameters for the island finder were determined by optimizing the rate of discovery of simulated regions (see below).

Core detection: cores were detected as an ARD except that a TimEX difference threshold of 0.1, a maximum gap size of 50,000 bp and a minimum island size of 50,000 bp were used in the GenPlay Island finder. Statistical significance was determined as above.

### G4 analysis

G4s were called using the Quadparser 2 software using the default parameters^34^.

### Intermolecular G4

iG4 were called on a Linux workstation using the following regular expression: \(CCC\|GGG\)[A-Z]\{1,7\}\(CCC\|GGG\)[A-Z]\{1,7\}\(CCC\|GGG\)[A-Z]\{1,7\}\(CCC\|GGG\), which finds all the iG4 and the majority of G4s. G4s were then subtracted from the results to obtain a list of all iG4s.

To determine if iG4 regulate initiation of replication, we defined iG4s as sequences that contain four consecutive triplets of either Cs or Gs separated by no more than seven nucleotides and searched the genome for all such sequences. We found that there were 485,926 iG4s in addition to the 361,000 G4s in the human genome and that about 10% of the iG4-forming sequences were also G4-forming sequences. Analysis of the overlaps between the origins of replication and iG4-forming sequences revealed that 50.5% of the origins of replication that we detected were associated with iG4s while 42% were associated with G4 (Fig. 4a). Approximately 62.9% of origins of replication were associated with either a G4 or an iG4. Permutation experiments revealed that the observed/expected ratio was similar for G4s and iG4s and that these associations were highly statistically significant (permutation *P* value <0.001 in all cases). Origins of replication are therefore as strongly associated with iG4-forming sequences as with G4-forming sequences.

To determine if iG4s regulate the origin efficiency, we computed the number of iG4s in the allele-biased origins of replication and compared this with origin efficiency. A total of 191 pairs of allelic origins contained different number of iG4s because of the presence of SNPs or indels. The allele containing the most iG4s was the most efficient origin in 87 cases and the least efficient in 104 cases (Fig. 4b), suggesting that the presence of iG4-forming sequences did not affect origin efficiency in any detectable manner. As above, we repeated the analysis focusing on the origins that contained only a single iG4 or only a single iG4 and no G4. As shown in Fig. 4b, no significant differences were found. We conclude from this analysis, that iG4s do not regulate initiation efficiency.

### GC-content and G-content analysis

GC-content for the comparison of G4-forming and non-forming 30-mers was calculated on the entire length of the studied regions. G-density in Fig. 6 and Supplementary Figs 5 and 6 was calculated in 50-bp moving windows with a window interval of 1 on the reference strand.

### Comparison of G4-forming and non-G4-forming sequences

To determine if the association between the origins of replication and G4-forming sequences was secondary to enrichment in GC-rich regions, we excluded all G4- and iG4-forming regions from the hg19 genomic sequence and selected random regions with the same length distribution as G4 and iG4-forming sequences. A set of ∼38 million unique control regions of the same length distribution as G4-forming sequence was created by repeatedly shuffling regions and excluding G4- and iG4-forming sequences. The GC-content of each region was then calculated and the distribution of GC-content was compared with that of the G4-forming regions. As shown in Supplementary Fig. 6, the two distributions were substantially different because the G4-forming regions had a high GC-content. To determine whether the origins of replication were associated with non-G4-forming sequences, we sampled these 38 million background non-G4-forming sequences to generate 100 distributions of 10,000 sequences with the same length and GC-content distribution as the G4-forming sequences and calculated the overlap with origins of replication.

### 30-mers with or without quadruplex-forming potential

We computed the list of all 30-mers (roughly the average size of G4 regions) in the genome with a sufficiently high GC-content to potentially harbour either a G4 or an iG4 secondary structure and created sublists of the 30-mers that contained or did not contain G4- and iG4-forming sequences. We then overlapped these lists with the list of origins of replication, calculated the average GC-content of all 30-mers containing G4- and iG4-forming sequence and found that it was equal to 71%. To determine whether the GC-content or the presence of G4- or iG4-forming sequences was the feature most strongly associated with origins of replication, we created a sublist of all 30-mers that could not form G4 or iG4 but that had a GC-content higher than 71% and calculated the overlap with the origins of replication. Fifty-seven per cent of these high GC-content 30-mers overlapped with an origin of replication, a higher percentage than the G4-forming 30-mers (Fig. 5b). We conclude that GC-rich 30-mers that do not contain G4- or iG4-forming sequences are more strongly associated with origins than similar 30-mers with this potential.

### Analysis of DNA skew

G-content histograms in Fig. 6a and Supplementary Fig. 3 were generated using the indicated number of biggest NS peaks defined as 100-bp regions centred on the peak summit. The G frequency was calculated as the frequency of G per 100 bp.

The G-frequency plot in Fig. 6b was generated using the top 3,000 NS peaks defined as 1 kb region centred on the summit of the peak. G frequency was calculated over 50 bp sliding windows using a step of one base pair. Subpeaks with a G frequency lower than the C frequency were reverse complemented. A K-means supervised clustering algorithm was then applied specifying four clusters.

Summary G/C skew plots (Fig. 6c and Supplementary Fig. 4) were generated using the indicated number of biggest NS peaks defined as 5-kb regions centred on the summit of the peak. G/C skew was then calculated as (G−C/G+C) on 50-bp sliding windows using a step of 1 bp. Summits with a G frequency lower than the C frequency in the 1-kb central window were reverse complemented to generate the adjusted GC skew. Adjusted G/C skew was then calculated on the stratified peaks by averaging the skew at each position. Summary A/T skew plots were generated in an analogous manner.

Bar plots in Fig. 6d were generated as follows. G/C skew was calculated as (G−C/G+C) in 50-bp windows. A/T skew was calculated as (A−T/A+T) in 50-bp widows. Genome-wide profiles of G/C skew and A/T skew were then calculated at 1-bp resolution in 50-bp windows using an R script. Regions with positive and negative skews were separated into two independent tracks using standard GenPlay filters. Positively skewed regions (G−C>0 on the plus strand) larger than about 400 bp were called using GenPlay Island finder after binning the 1-bp resolution track at 25 bp. Parameters for the island finder were (window value=0.375; gap=4; island score=16; island length=15). The track containing the regions with a negative skew (G−C<0 on the plus strand) was then multiplied by −1 to convert the negative skew into a positive skew. The skewed regions on this modified track were then called as described above. The called positively and negatively skewed regions were then combined to create a track that contains all regions with a skewed G or C distribution. This combined track was then intersected with tracks containing the origins of replication and permutations were performed to evaluate statistical significance.

### Triplex analysis

Potential intramolecular triplex patterns were analysed using the Bioconductor package Triplex (v.1.4.0)^58^. Analyses performed either on the 164,000 peaks or on the subpeaks yielded very similar results. Analysis was performed using either the default parameters or a minimum score of 15 and a *P* value of 1. Filtering of the triplexes most likely to form using a *P* value=0.05 led to very similar conclusions to considering all potential triplex-forming sequences.

### Motif search

Motif searches were performed using the MEME^59^ and the Homer application. Once motifs were found, they were mapped genome-wide using PWMScan^60^

### Statistics

Permutation analyses were performed using R scripts. Chromosomal intervals were randomized using the shuffleBed function of Bedtools (v. 2.20.1)^61^. Depending on the size of the file, 1,000–10,000 iterations were performed to define the expected values. *P* values were calculated as the number of observations obtained with shuffled data that were above the observed value with the real data / total number of iterations. Most statistics were performed in R or with perl scripts.

## Additional information

**Accession codes:** The fastq, aligned files (.bam) and several process files have been deposited to the NCBI Gene Expression Omnibus (GEO) under accession number GSE61972. Files containing the G4, iG4, Triplex and nucleotide genome-wide data can be accessed at http://genplay.einstein.yu.edu/library/Human/hg19/Non_B_DNA/. The entire data set can be visualized as a GenPlay Project at http://genplay.einstein.yu.edu/library/projects/Allele_Specific_NS_Sequencing_FNY01_2_2.

**How to cite this article:** Bartholdy, B. *et al*. Allele-specific analysis of DNA replication origins in mammalian cells. *Nat. Commun.* 6:7051 doi: 10.1038/ncomms8051 (2015).

## Supplementary Material

Supplementary Information Supplementary Figures 1-6

## Figures and Tables

**Table 1:** Overlap between origins and genomic features.

	**All origins (%)**	**Non-analysable origins (%)**	**Non-analysable origins (resampled; %)**	**Analysable**		
				**All analysable origins (%)**	**Allele-biased origins (%)**	**Non-allele-biased origins (%)**
**All**	164,327	141,483	22,562	22,844	1,992	20,852
**G4**	69,394 (42.2)	56,486 (39.9)	13,279 (58.85)	12,908 (56.5)	1,157 (58.1)	11,751 (56.4)
**TSS**	16,485 (10.0)	14,559 (10.3)	3,098 (13.73)	1,926 (8.4)	183 (9.2)	1,743 (8.4)
**CpGI**	23,903 (14.5)	20,694 (14.6)	4,792 (21.24)	3,209 (14.0)	305 (15.3)	2904 (13.9)
**Imprinted**	9,497 (5.8)	7,845 (5.5)	1,700 (7.53)	1,652 (7.2)	146 (7.3)	1506 (7.2)
**LINE**	42,343 (25.8)	34,240 (24.2)	6,616 (29.32)	8,103 (35.5)	749 (37.6)	7354 (35.3)
**SINE**	60,095 (36.6)	48,961 (34.6)	9,968 (44.18)	11,134 (48.7)	927 (46.5)	10207 (48.9)
**LTR**	25,878 (15.7)	21,324 (15.1)	3,388 (15.02)	4,554 (19.9)	453 (22.7)	4101 (19.7)

Analysable origins are origins that contain at least 50 SNP-containing reads and that could therefore be analysed in an allele-specific manner. In column 4, the non-analysable origins were resampled to match the peak height distribution of the analysable origins. The absolute number (and the percentage) of origins that overlap with each feature is indicated. The percentage is calculated as (100 × no. of the origin overlapping a genomic feature/total no. of origin in that subset). The analysable origins are richer in G4, LINE, SINE and LTR than the non-analysable origins. Resampling the non-analysable origins to create a new subset of origins with the same peak height distribution as the analysable origins largely eliminates the differences in G4, imprinted genes, LINE and SINE content between the analysable and non-analysable origins. Analysable origins are therefore representative of all relatively strong origins that we detected (see text).

**Table 2:** Sequence and position of 14 G4s located in origins containing a unique polymorphic G4.
