# Supplementary material for: Allele-specific analysis of DNA replication origins in mammalian cells
Source: Nat Commun. 2015 May 19;6:7051. doi: 10.1038/ncomms8051 (PMC4479011; doi:10.1038/ncomms8051)
Supplement: Supplementary Information — Supplementary Figures 1-6 [file ncomms8051-s1.pdf]

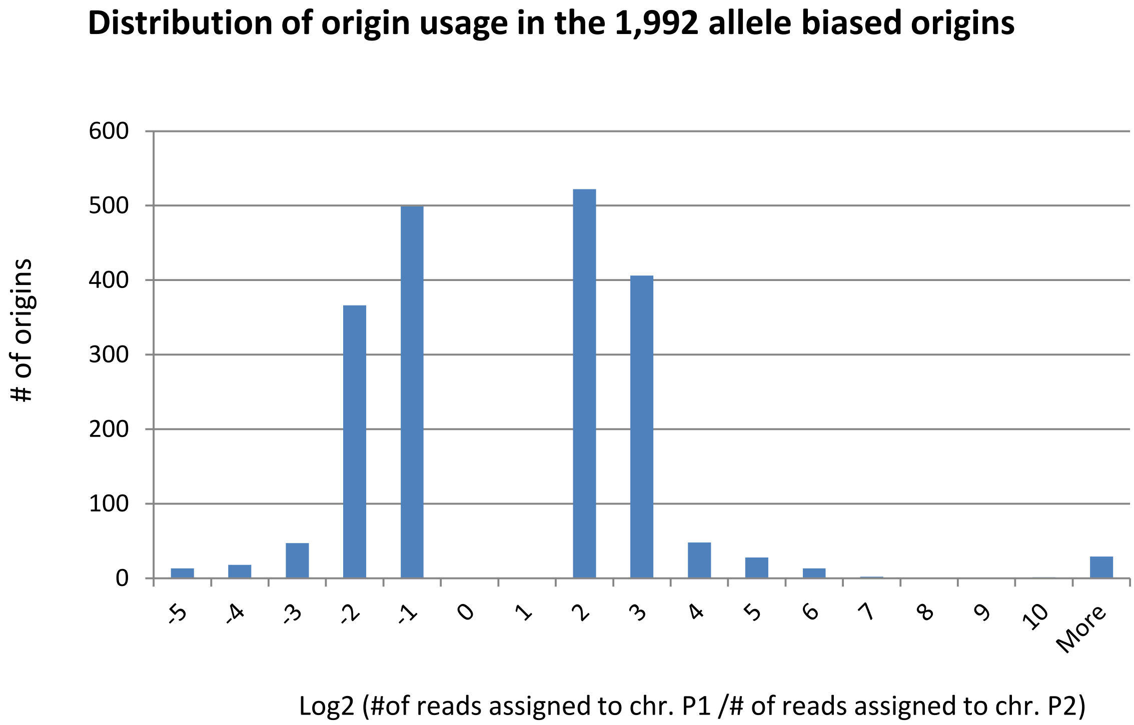

Supplementary Figure 1: **Distribution of origin usage in the 1,992 allele-biased origins**. The x-axis represents the Log_2 (number of reads assigned to chr P1 / number of reads assigned to chr P2). The y-axis represents the frequency.

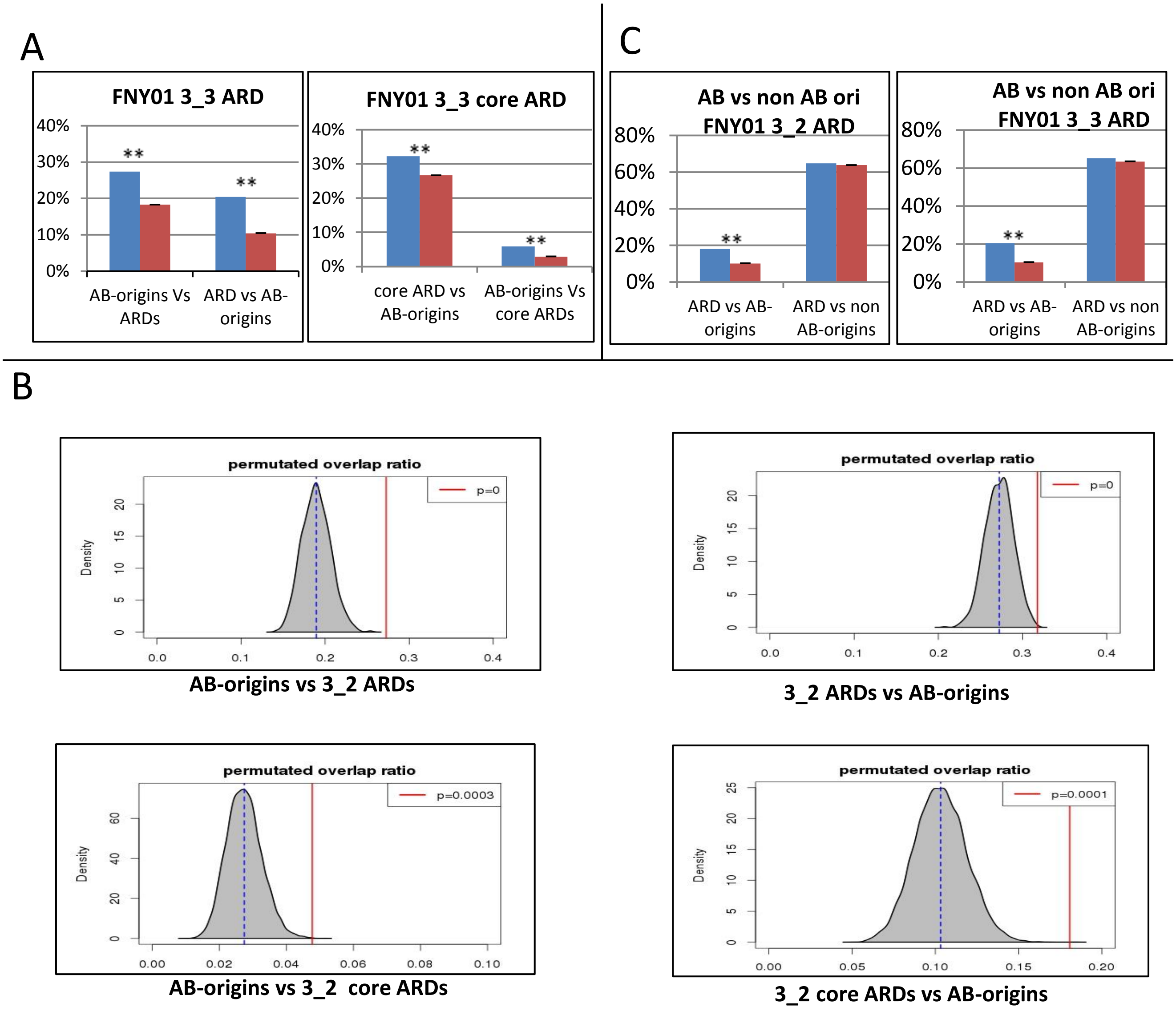

Supplementary Figure 2: ARDs and core ARDs are highly enriched in allele-biased origins of replication. A: ARDs and core ARDs of individual FNY01_3_3 are enriched in allele biased origins of replication. Left histogram: percent ARDs that contain allele-biased origins. Right histograms: percent core ARDs that contain allele-biased origins. Blue bars: observed values; red bars: expected values; error bars represent s.e.m of permutations; AB-origins: = allele biased origins. Stars indicate that the differences between observed and expected values were significant. B: Density plots illustrating the results of permutation experiments to determine the expected overlap of ARDs with allele-biased origins caused by chance alone. The location of the allele-biased origins was randomized 10,000 times and overlapped with the ARDs. The x-axis represents the percent overlaps. The red line illustrates the observed results. The area under the curve summarizes the results of the 10,000 simulation. The y-axis represents the kernel density of the frequency of overlap. P.values were calculated as the ratio of the number of times the percent overlap was greater than the observed values/ by the total number of permutations. C: Histograms summarizing the enrichments of ARDs in allele-biased (AB) and non-allele-biased origins for FNY01 3_2 (left) and FNY01 3_3 (right). ARDs are enriched in allele biased (p <1.E-04) but not in non-allele-biased origins. Blue bars: observed values; red bars: expected values. Expected values and p values were calculated as above. Stars indicate that the differences between observed and expected values were significant. Error bars represent s.e.m of permutations

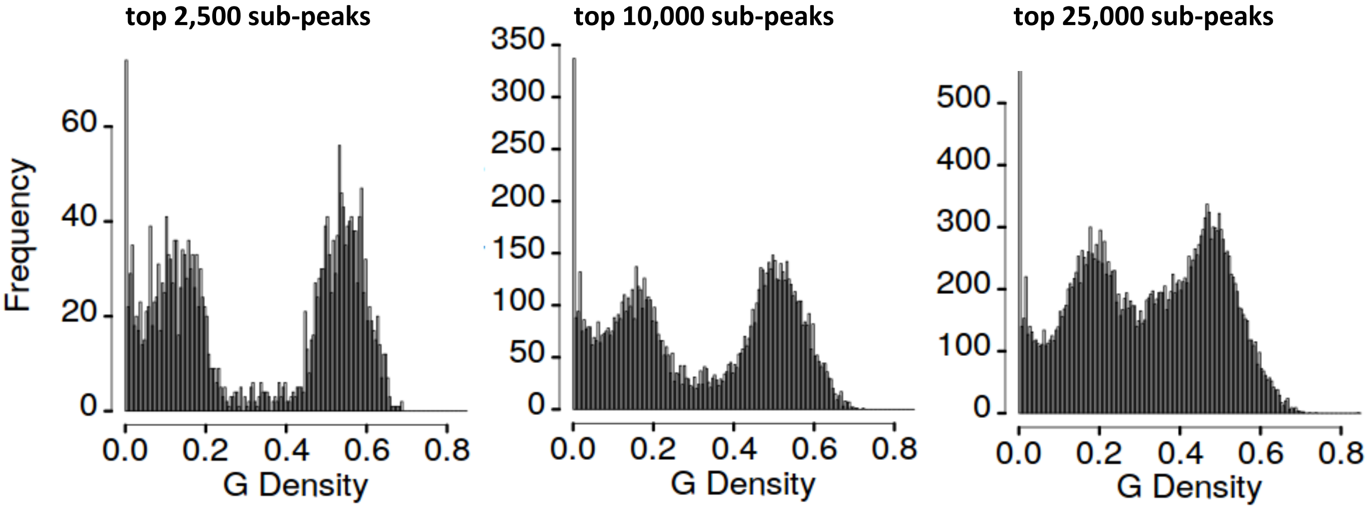

Supplementary Figure 3: **Histograms illustrating G-density in origin sub-peaks**. The bimodal distribution reflects the fact that the G-rich strand is either on the plus or minus strands. G-density was calculated on 100 bases windows. Highly efficient origins have a highly biased G- and C-density. Weaker origins exhibit a smaller bias.

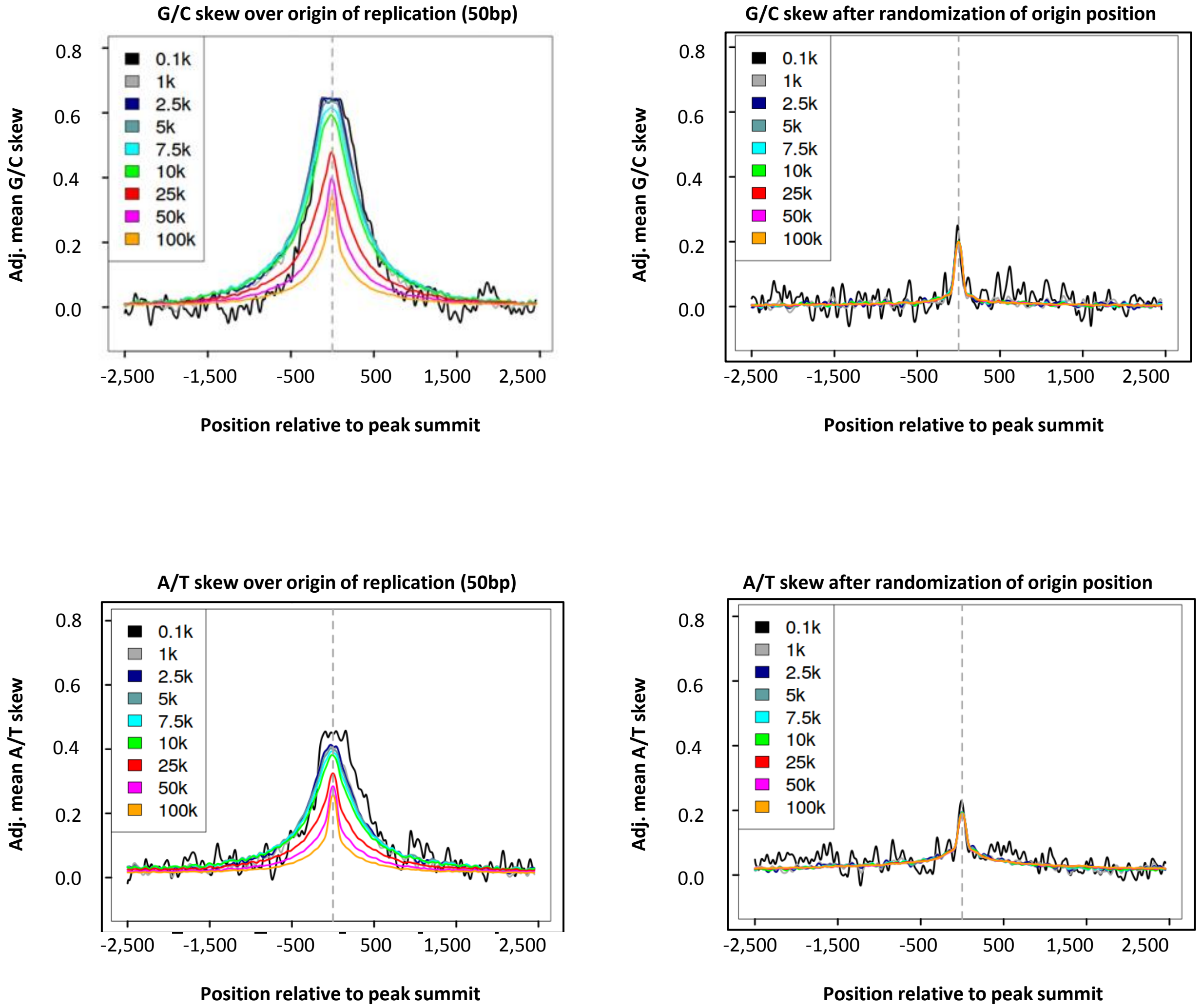

Supplementary Figure 4: X-Y scatter plots of G/C and A/T skew in origin sub-peaks stratified by origin efficiency. The black line represents the adjusted G/C skew of the top 100 most efficient origins (0.1k). The grey line the adjusted G-density the top 1000 origins (1k) etc… G/C skew was adjusted by reverse complementing the origins in which the G-rich strand was the minus strand (see methods). Histograms on the right are control histograms in which the positions of the origin sub-peaks were randomized. The small peak in the center is caused by the reverse complementation of the origins that exhibit a low G-density (or A-density) on the minus strand. Highly efficient origins contain large 200-500bp skewed regions. The size of the skewed region and the amount of skew progressively decrease in the less efficient origins.

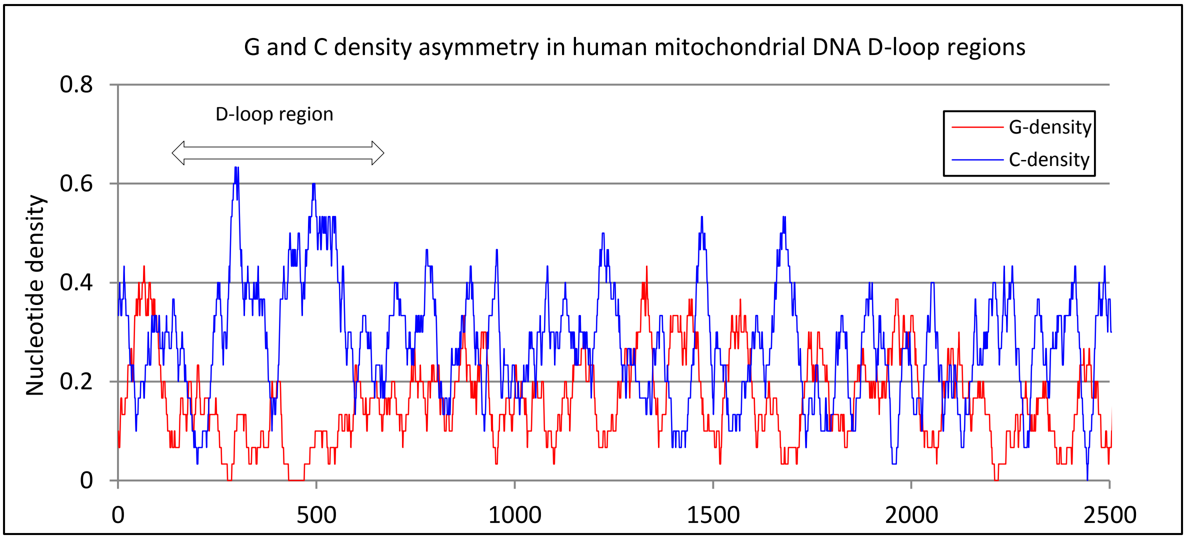

Supplementary Figure 5: Human mitochondrial DNA D-loop region which contains the major origin of replication O_H is profoundly G/C skewed. G and C-density were calculated in 50 base pair windows with a step of 1 using EMBOSS freak program. The sequence of human mitochondrial DNA was obtained from GenBank (NC_012920).

GC content distribution in G4-forming regions

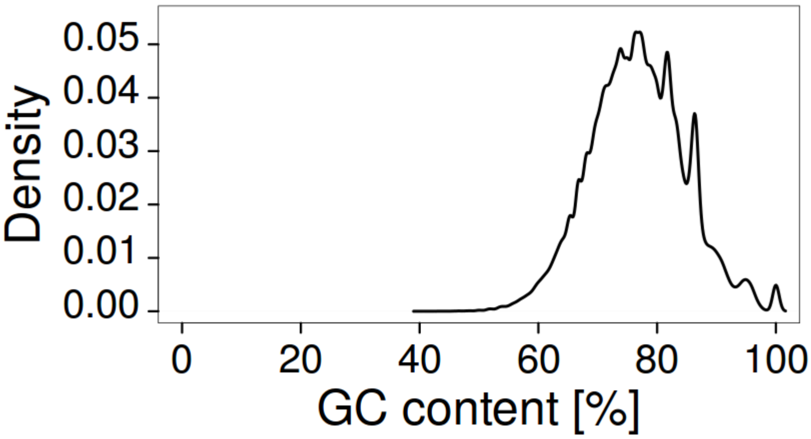

GC content distribution in non-G4-forming regions

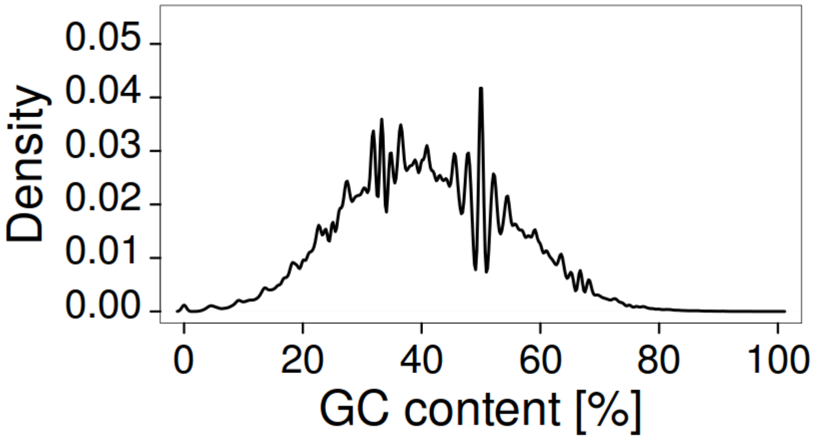

Supplementary Figure 6: Density plot illustrating the GC-content of G4-forming 30-mers as compared to a control set of 30-mers depleted of G4 and iG4-forming sequences with same length distribution.
